# Tissue-Based Biomarkers for Fluorescence-Guided Surgery of Pancreatic Ductal Adenocarcinoma: A Systematic Review

**DOI:** 10.3390/cimb48070717

**Published:** 2026-07-14

**Authors:** Ahmed Boalot, Amira Younes, Callie-Jo Woodward, Afnan Alelaimi, Ferhat Arabaci, Donghyun Lee, Claire H. Ozber, Michal Heger, Yazan S. Khaled

**Affiliations:** 1 Leeds Institute of Medical Research, University of Leeds, Leeds LS9 7TF, UKc.h.ozber@leeds.ac.uk (C.H.O.); 2 Applive Research Laboratories, Division of Medicine, Jiaxing University, Jiaxing 314001, China; michal.heger@photonanomedicine.com; 3 The Pancreato-Biliary Unit, St. James’s University Teaching Hospital, Leeds LS9 7TF, UK

**Keywords:** tissue biomarkers, targeted molecular imaging, biomarker selection, pancreatic ductal adenocarcinoma

## Abstract

Pancreatic ductal adenocarcinoma (PDAC) is an invasive cancer with poor survival outcomes and limited treatment options. This systematic review aimed to identify and evaluate tissue-based biomarkers with potential applications in fluorescence-guided surgery (FGS), an approach that enables real-time intraoperative tumour visualisation to improve surgical precision. A systematic review was conducted in accordance with PRISMA guidelines and registered on PROSPERO (CRD420251056295). A comprehensive literature search was conducted across MEDLINE, Embase, and Cochrane Library databases from 1980 to January 2026 and was updated in March 2026. The EU Clinical Trials Registers were also searched for biomarker-targeted clinical trials. The TASC-T (Theranostic Target Selection Criteria) scoring system was used to assess biomarker suitability. EGFR, MSLN, CEA, and integrin αvβ6 scored highest, reflecting their strong potential as theranostic targets. MUC1 and uPAR also demonstrated high positivity and accessibility for imaging and therapy. While CA19-9 is a widely used serum biomarker, its role as a tissue target appears limited because of its high expression in benign conditions. EGFR and αvβ6 are both undergoing evaluation in phase II clinical trials and have emerged as promising targets for FGS in PDAC. This review highlights the need for standardised protocols and collaborative research to expand the clinical utility of these biomarkers. The methods and scoring criteria used in this review provide a transferable framework for identifying tissue biomarkers in other cancers and disease states.

## 1. Introduction

Pancreatic ductal adenocarcinoma (PDAC) is a fatal disease with a 5-year survival rate of 8–13% [1]. Complete surgical resection remains the only curative option for PDAC but is seldom achieved, with positive resection margins (R1) reported in up to 70% of cases [2,3,4]. Overall survival (OS) in unresectable disease, with or without palliative chemotherapy, remains poor [5]. There is an urgent clinical need to improve outcomes by identifying novel diagnostic markers and advancing targeted therapies. This includes the development of innovative strategies such as nanomedicine-based technologies and predictive markers to optimise therapeutic responses [6,7].

PDAC resection aims to achieve complete oncological clearance while minimising peri-operative morbidity and mortality. One of the major limitations of current pancreatic cancer surgery is the lack of real-time intraoperative disease identification. This limits the surgeon’s ability to confirm complete tumour resection, detect involved lymph nodes (LNs), and identify occult micrometastatic disease during the operation. As a result, residual malignant tissue may remain undetected, contributing to incomplete tumour removal and subsequent disease recurrence [8]. This challenge is further compounded by the limited sensitivity of current pre-operative imaging modalities for detecting small lesions, occult metastases, and microscopic nodal disease [9,10]. Accordingly, there is an unmet clinical need for intraoperative imaging strategies that can enhance cancer detection and improve complete resection rates in PDAC. Fluorescence-guided surgery (FGS) offers a potential solution by enabling real-time visualisation of malignant tissue, involved LNs, and occult metastatic deposits during surgery. Rather than applying a uniformly radical surgical approach, fluorescence-guided techniques may support a more personalised operative strategy in which the extent of resection is tailored to the intraoperative disease burden. This approach may help minimise unnecessary tissue removal while improving the likelihood of achieving negative resection margins. Intraoperative fluorescence imaging may also help identify LN micrometastases that are not detected by standard imaging techniques [11]. Furthermore, it may assist surgeons in distinguishing viable tumour tissue from post-treatment fibrosis or inflammatory changes, an increasingly relevant challenge with the growing use of neoadjuvant chemotherapy. Fluorescence-guided detection may also be valuable during staging laparoscopy, where improved visualisation of peritoneal metastases, liver metastases, and extra-pancreatic LNs could refine staging accuracy and guide surgical decision making.

The effectiveness of FGS depends on the availability of tumour-specific biomarkers that can be used as molecular targets for fluorescent probes, fluorophore-conjugated ligands, antibodies, peptides, or other targeted delivery systems [12,13,14]. These approaches aim to generate sufficient tumour-to-background contrast by selectively binding to, or accumulating within, malignant tissue while limiting non-specific signal in surrounding normal structures. Therefore, identifying reliable PDAC-associated tissue markers is essential for the development of targeted fluorescence imaging strategies that could improve intraoperative cancer detection and guide surgical decision-making. 

The efficacy of antibody-based targeted fluorescence imaging in PDAC has been shown in two recent clinical trials. In phase I clinical trials, fluorescently labelled antibodies against epidermal growth factor receptor (EGFR) and carcinoembryonic antigen (CEA) were used to guide surgical resection of PDAC [15,16]. The authors reported that intraoperative fluorescence enhanced visualisation of primary tumours compared to surrounding normal tissue. These studies suggest that molecularly targeted fluorescence imaging can improve visualisation of PDAC tissue compared with adjacent normal tissue. However, the number of clinically evaluated targets remains limited, and further work is required to identify additional biomarkers with sufficient tumour specificity, expression consistency, and clinical relevance for FGS. Determining the status of such markers preoperatively could refine current staging criteria and improve the selection of patients for personalised treatments, as has been performed, for example, in biliary cancers [17,18,19]. Molecular analysis offers a valuable source of clinically relevant biomarkers, as tumours with similar clinicopathological features often exhibit molecular differences that drive variations in clinical outcomes. A recent systematic review on the use of active targeting nanoparticle delivery systems in targeted cancer therapy found no translational studies focusing on PDAC [20]. This highlights the urgent need to combine research efforts to identify specific tissue biomarkers that enable precision diagnostics and therapeutics for this lethal cancer.

In this study, we conducted a systematic review of the literature to identify tissue-based biomarkers suitable for active tumour targeting in PDAC, with particular emphasis on their potential application in FGS. By identifying candidate biomarkers that could support targeted fluorescence imaging, this review aims to provide a framework for improving intraoperative tumour visualisation, guiding surgical decision making and supporting future personalised approaches in PDAC management. Additionally, the methods outlined here could serve as a framework for identifying theranostic biomarkers in other cancers and diseases.

## 2. Materials and Methods

### 2.1. Systematic Search Strategy for Tissue Biomarkers in PDAC

This systematic review was conducted and reported in accordance with the Preferred Reporting Items for Systematic Reviews and Meta-Analyses (PRISMA) 2020 statement [21]. This is a new systematic review and does not constitute an update to any previously published review. To our knowledge, no prior systematic review has specifically examined tissue-based biomarkers or molecular targets in PDAC within the context of active tumour targeting for fluorescence-guided surgery using a structured assessment framework. The protocol was prospectively registered on PROSPERO (registration number: CRD420251056295) in May 2025. Electronic searches were performed using MEDLINE/PubMed, Embase, and the Cochrane Library from 1980 to January 2026. The search strategy was designed to identify studies reporting tissue-based biomarkers or molecular markers in PDAC with potential relevance to tumour targeting and FGS.

For MEDLINE/PubMed, both Medical Subject Headings (MeSH) and free-text terms were used. MeSH terms were used where available, while free-text terms were searched in titles, abstracts, and all fields to capture recently published articles and records not yet fully indexed. Quotation marks were used only for exact phrases, such as “pancreatic ductal adenocarcinoma”, “pancreatic cancer”, “molecular marker”, and “tissue biomarker”. Individual keywords were not placed in quotation marks unless they formed part of an exact phrase.

The search combined three main concepts: pancreatic cancer/PDAC, tissue-based biomarkers or molecular markers, and tumour-targeting relevance. The PubMed search strategy included combinations of the following terms: (“carcinoma, pancreatic ductal” [MeSH Terms] OR “pancreatic ductal adenocarcinoma” OR “pancreatic adenocarcinoma” OR “pancreas adenocarcinoma” OR “pancreatic cancer” OR “pancreas cancer” OR “pancreatic tumour” OR “pancreatic tumor” OR “pancreas tumour” OR “pancreas tumor” OR “pancreatic malignancy” OR “pancreas malignancy” OR “pancreatic neoplasm” OR “pancreas neoplasm” OR PDAC) AND (“tissues”[MeSH Terms] OR tissue OR tissues OR “tissue-based”) AND (“biomarkers”[MeSH Terms] OR biomarker OR biomarkers OR “tissue biomarker” OR “molecular marker” OR “molecular markers” OR antigen OR receptor OR target OR targets). Where supported by the database, truncation was used to capture singular, plural, and related word forms. For example, biomarker* was used to retrieve “biomarker” and “biomarkers”, and marker* was used to retrieve “marker” and “markers”. Where truncation was not supported, singular and plural forms were entered separately. The search strategy was adapted for each database according to its specific syntax. MeSH terms were used only for MEDLINE/PubMed; equivalent database-specific subject headings and free-text terms were used where applicable for Embase and the Cochrane Library.

The original search string was revised to remove duplicated terms. In particular, repeated use of “tissue” and “biomarker” was removed because these repetitions did not alter the search logic but reduced clarity. The word “tissue” was retained only once within the tissue biomarker concept group to reflect the focus of this review on tissue-based markers relevant to active tumour targeting. The EU Clinical Trials Register and ClinicalTrials.gov were also searched using the terms “pancreatic cancer” OR “pancreatic ductal adenocarcinoma”, together with clinically available monoclonal antibodies and targeted agents relevant to PDAC. 

**Figure 1:**
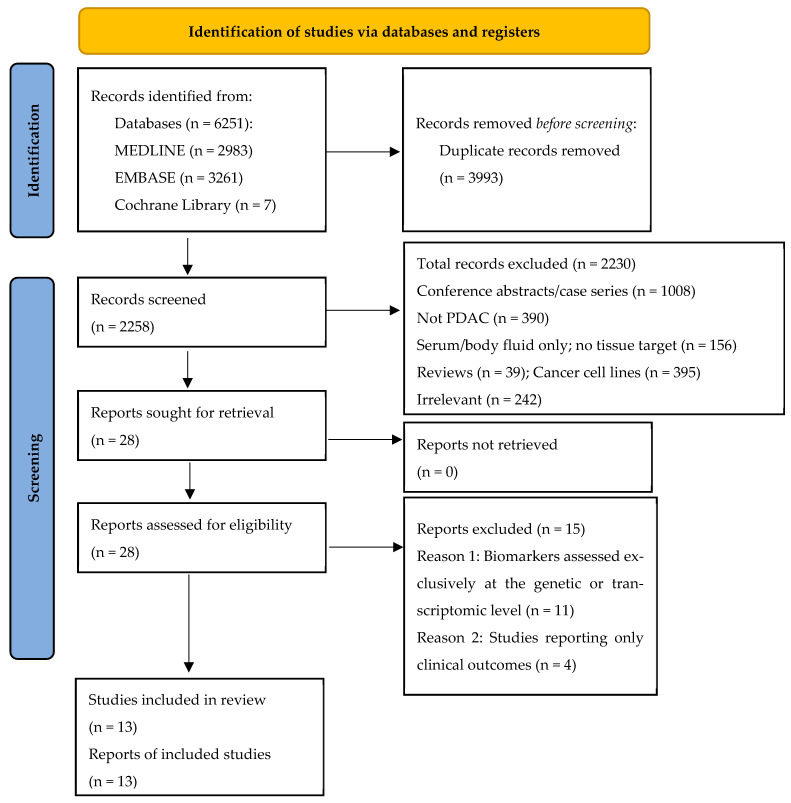
PRISMA 2020 flow diagram illustrating the study selection process. Records were identified through searches of three electronic databases: MEDLINE/PubMed (n = 2983), Embase (n = 3261), and the Cochrane Library (n = 7). A total of 6251 records were identified, of which 3993 duplicates were removed prior to screening. Of 2258 records screened at the title and abstract stage, 2230 were excluded for the following reasons: conference abstracts or case series (n = 1008), cancer cell lines (n = 395), wrong tumour type (not PDAC; n = 390), irrelevant to the review question (n = 242), serum or body fluid biomarkers only with no tissue-based target assessed (n = 156), and review articles (n = 39). Twenty-eight full-text reports were assessed for eligibility, of which 15 were excluded because biomarkers were assessed exclusively at the genetic or transcriptomic level without protein-level tissue expression data (n = 11) or because reports contained only clinical outcomes without extractable tissue biomarker expression or target-localisation data relevant to imaging (n = 4). Thirteen studies were ultimately included in the review. The flow diagram was produced in accordance with PRISMA 2020 reporting guidelines.

An updated search was conducted in March 2026 to identify newly disclosed data relevant to this review. Eligibility was limited to English-language full-text primary research articles. The TASC-T scoring system was used to identify biomarker characteristics most relevant to PDAC tumour targeting. A total of 6251 records were identified during the search. The PRISMA flow diagram is shown in Figure 1.

Title and abstract screening and full-text eligibility assessment were performed by four reviewers (CJW, AA, AB, and AY) according to a predefined protocol. Data extraction was performed by two reviewers (CJW and AA) using a standardised data extraction form. Extracted data were subsequently cross-checked for accuracy and completeness by two reviewers (AB and AY). Any discrepancies or uncertainties were resolved through discussion, and unresolved disagreements were adjudicated by the corresponding author (YSK). Formal inter-reviewer agreement statistics were not calculated prospectively; however, the consensus process was used to verify all extracted data before final inclusion in the review.

Clinical trial registry records were screened using the same eligibility criteria as the published literature search, where applicable. Only trials involving PDAC or pancreatic cancer and evaluating relevant tissue-based molecular targets, monoclonal antibodies, targeted imaging agents, or target-directed interventions were retained.

### 2.2. Selection Criteria

Title and abstract screening led to the exclusion of 2230 records. All records and full-text reports were screened using the inclusion and exclusion criteria presented in Table 1.

### 2.3. Data Curation

A narrative analysis of biomarkers in PDAC was conducted due to heterogeneous outcomes across studies. Data extracted included study characteristics, biomarker details (type, tissue specificity and expression levels), analysis methods (IHC, tissue microarrays (TMA), reverse transcriptase polymerase chain reaction (RT-PCR)), and key outcomes such as sensitivity and specificity. 

### 2.4. Quality Assurance

Quality assurance was implemented using the REMARK (Reporting Recommendations for Tumor Marker Prognostic Studies) assessment [22] to evaluate reporting quality, methodological transparency, and potential risk of bias (Supplementary Table S1). The adapted REMARK assessment evaluated the following thirteen domains: patient cohort description, eligibility criteria, clinicopathological data, tissue specimen source and handling, biomarker assay method, antibody and reagent details, scoring and cut-off method for biomarker positivity, blinding or independent assessment, outcome or target validation endpoint, statistical analysis, confounder adjustment, completeness of results, and acknowledgement of limitations or sources of bias. Each domain was rated as Yes = clearly reported/low concern (score 2), Partial = incompletely reported/some concern (score 1), No = not reported/high concern (score 0), or N/A = not applicable (excluded from the denominator). The percentage of fulfilled items was calculated as (total score/maximum applicable score) × 100. Overall risk-of-bias judgement was assigned using equal interval thresholds: Low risk > 67%, Moderate risk 34–67%, and High risk < 34%. Conservative ratings were applied: where critical methodological details were absent from the main text and deferred to Supplementary Materials, the domain was rated Partial rather than Yes.

## 3. Results

### 3.1. Literature Search

A systematic search of the literature was conducted following the PRISMA guidelines. A total of 6251 records were identified through the database search. Following full text review, 13 studies met the inclusion criteria and were included in the final analysis, as shown in Table 2. The characteristics of their respective tissue biomarkers in PDAC are presented in Table 3. Although 6251 records were initially identified, only 13 studies met the final eligibility criteria. This reflects the broad initial search strategy and the specific focus of the review on tissue-based PDAC biomarkers relevant to active tumour targeting and FGS. Many excluded records investigated serum or body fluid biomarkers, non-PDAC malignancies, prognostic markers without tissue-targeting relevance, non-human models, or cell line-only experiments. Cell line-only studies were excluded from the main analysis unless supported by validation in primary human PDAC tissue, as the purpose of this review was to prioritise biomarkers with direct tissue relevance for intraoperative fluorescence imaging.

A total of 23 tissue biomarkers were identified in these studies. Data on biomarker expression, co-expression, sensitivity, specificity, and association with lymph node (LN) infiltration were extracted from the included studies, providing a comprehensive understanding of their potential as fluorescent imaging targets for PDAC.

### 3.2. Risk of Bias/Quality Assessment

The methodological quality and potential risk of bias of the 13 included studies were assessed using an adapted REMARK framework. Overall, eight studies were judged to have a low risk of bias, and five studies were judged to have a moderate risk of bias. No study was judged to be at high risk of bias. Studies at low risk of bias generally provided clear reporting of the patient cohort, eligibility criteria, tissue handling, biomarker assay methodology, scoring or cutoff criteria, endpoints, and statistical analysis. Studies at moderate risk of bias commonly had incomplete reporting of cohort selection criteria, clinicopathological characteristics, assay methodology, reagent details, blinding procedures, or study limitations, often because key methodological details were deferred to Supplementary Materials not reproduced in the main text. Across the included studies, the most frequent methodological concerns related to incomplete reporting of blinding or independent review, variable reporting of reagent details, inconsistent definition of biomarker positivity cutoff thresholds, and limited acknowledgement of study limitations. The full study-level REMARK-based assessment, including domain-level ratings, total scores, and percentage of fulfilled items, is provided in Supplementary Table S1.

### 3.3. Tumour-Targeted Fluorescent Agents in Clinical Trials

To date, four targeted fluorophore-conjugated agents have entered clinical evaluation in patients with PDAC. Table 4 summarises the potential of these biomarkers for imaging applications in PDAC, with supporting evidence from key clinical studies.

A review of the EU Clinical Trials Register (https://www.clinicaltrialsregister.eu/ (accessed on 26 April 2026)) and ClinicalTrials.gov (https://clinicaltrials.gov/ (accessed on 26 April 2026)) identified several targeted molecular imaging agents currently undergoing clinical investigation for PDAC-associated cell surface biomarkers. These include cRGD-ZW800-1 (NCT05518071; recruitment completed, results pending), which targets integrins (αvβ3, αvβ5, αvβ6); panitumumab-IRDye800 (NCT03384238; recruiting) and anti-EGFR-IR800CW (NCT06395337; recruiting), both targeting EGFR; and DOTA-anti-CEA (NCT06168552; recruiting), targeting CEA.

### 3.4. Selection of Biomarkers

A scoring system for the selection of potentially targetable biomarkers for imaging in colorectal cancer (TArget Selection Criteria or TASC score) has been reported by van Oosten [38]. However, no published scoring tools exist for selecting biomarkers that incorporate ligands in clinical trials or biomarkers that exhibit minimal or no presence in normal tissue. To address this need, a modified version of the TASC score [38], called the TASC-Theranosis score (TASC-T), was adapted for evaluation of each biomarker [39]. The 23 selected candidate biomarkers that were presented in Table 2, Table 3 and Table 4 were evaluated using the TASC-T scoring criteria outlined in Table 5. Points of varying weight were awarded for the following criteria: extracellular location, >20% positivity in tumour tissue, low expression in normal pancreatic tissue, percentage positivity or upregulation in PDAC tissue, prior use as an imaging or theranostic biomarker (in preclinical or clinical studies), and the availability of a clinically tested ligand for targeting. Biomarkers automatically received 4 points for exceeding 20% positivity in tumour tissues, as this was a selection criterion for inclusion in the study. The maximum achievable score was 22. Table 6 shows the TASC-T scoring for the bestperforming biomarkers. Any biomarker with a scoring of 17 or higher was considered strong candidates (similar to TASC score described by van Oosten et al. [38]). EGFR, MSLN, CEA, and integrin αvβ6 scored the highest, reflecting their strong potential as specific targets for FGS. MUC1, uPAR, and CA19-9 also showed high positivity and accessibility for targeting. 

### 3.5. Enumerated Biomarkers for Theranosis in PDAC

#### 3.5.1. CA19-9

Carbohydrate antigen 19-9 (CA19-9) is a membrane-bound glycan that is widely used as a clinical serum marker for PDAC detection and disease monitoring [44]. Its tissue overexpression ranges from 70–90% and correlates with disease progression and OS [35,44]. However, its specificity is limited due to expression in benign pancreatic conditions and inflammatory tissues, such as chronic pancreatitis (CP) and normal pancreatic tissue (NPT) [45,46,47]. Despite being FDA-approved for serum detection, its upregulation in non-neoplastic tissues reduces its utility for accurate diagnosis. TASC scoring highlights its imaging potential but notes limitations in distinguishing PDAC from benign processes.

#### 3.5.2. CEA (CEACAM-5)

Carcinoembryonic antigen (CEA), or CEACAM-5, is a membrane-bound adhesion molecule overexpressed in 70–85% of PDAC cases [11,24,28]. It plays a role in extracellular matrix adhesion, tumour motility, and inhibition of apoptosis [48,49]. Importantly, CEA is absent in NPT but shows moderate expression in CP, reducing its diagnostic specificity [34]. Studies report reduced expression after neoadjuvant therapy (NAT), which may affect its use in imaging and staging [11]. Co-expression of CEA with integrin αvβ6 enhances its potential as a dual target for PDAC imaging and detection of lymph node metastasis (sensitivity 83%, specificity 100%) [28].

#### 3.5.3. EGFR

Epidermal growth factor receptor (EGFR) is a transmembrane receptor tyrosine kinase involved in PDAC cell proliferation, metastasis, and angiogenesis [50]. EGFR overexpression in PDAC has been reported with considerable variability across studies. For instance, a study by Wu et al. indicated that EGFR was overexpressed in 7.7% to 100% of PDAC samples, depending on factors such as sample selection and methodology used for detection [51]. Moreover, studies have shown that EGFR overexpression is associated with advanced disease stages and poor differentiation, which are critical factors influencing patient survival [29]. For example, Huang et al. reported that EGFR overexpression correlates with metastasis and poor prognosis in PDAC patients [52]. This suggests that EGFR could serve as a biomarker for aggressive disease and a potential target for drug targeting [53,54] or therapeutic intervention. Given the high rates of EGFR overexpression in PDAC, targeting this receptor has become a focal point in developing therapeutic strategies. Erlotinib, an EGFR inhibitor, has been evaluated in clinical settings, with mixed results regarding its efficacy in improving patient outcomes [55]. The variability in EGFR expression among patients may contribute to the inconsistent responses observed in clinical trials [56]. Moreover, the combination of EGFR inhibitors with other therapeutic agents, such as gemcitabine, has been explored to enhance treatment efficacy. Studies have shown that combining these therapies can lead to improved outcomes in certain patient populations, particularly those with high EGFR expression [57]. TASC scoring identifies EGFR as one of the most promising imaging biomarkers (score of 21). Although targeting EGFR presents a promising therapeutic avenue, further research is needed to clarify its prognostic significance and to optimize treatment strategies that incorporate EGFR inhibitors.

#### 3.5.4. Integrin αvβ6

Integrins are cell membrane receptors found in epithelial tumours such as PDAC and colorectal cancer [58,59,60]. Overexpression of integrin αvβ6 in PDAC is linked to advanced disease and poorer survival, highlighting its role in disease progression [61]. Integrin αvβ6 is a cell membrane receptor overexpressed in 80–88% of PDAC tissues, mediating cell adhesion, migration, and tumour progression [62]. Steiger et al. (2017) found a high expression of integrin β6 on tumour cells in 88% of nearly 400 specimens of PDAC primaries and in virtually all metastases [63]. Its absence in normal pancreatic tissues and persistence in fibrotic tissue post-NAT make it a reliable target for imaging and therapy in PDAC [24]. As mentioned, dual targeting of integrin αvβ6 with CEA has shown improved sensitivity and specificity for LNM detection [24]. Preclinical studies highlight the potential clinical application of integrin αvβ6-targeted agents for fluorescence and acoustic imaging [64].

#### 3.5.5. MUC-1

Mucin-1 (MUC-1) is a highmolecularweight transmembrane glycoprotein overexpressed in 90–96% of PDAC tissues [23,32,65]. Altered glycosylation of MUC-1 enhances tumour invasiveness, metastatic potential, and resistance to chemotherapy [66]. Its overexpression correlates with aggressive biological behaviour and poor prognosis [23,67]. However, MUC-1 expression in NPT and downregulation following NAT pose challenges for its diagnostic specificity [11,23]. Despite these limitations, its high accuracy in NLM detection (high accuracy in positive LN detection and absent expression in negative LN) and consistent overexpression make it a strong candidate for PDAC imaging and molecular targeting [68]. 

MUC5AC and MUC16 also warrant brief consideration, although neither was prioritised over MUC1 using the TASC framework. MUC5AC is a pancreatic cancer-associated mucin with diagnostic and prognostic relevance; however, its suitability for FGS may depend on glycoform-specific expression, tumour cell localisation, and tumour-to-background contrast [69,70]. MUC16, also known as CA125, is biologically relevant because of its interaction with mesothelin, an axis implicated in pancreatic cancer invasion, migration and metastatic behaviour [71,72]. MUC16-targeted near-infrared probes have also been evaluated preclinically for pancreatic cancer FGS [73,74]. However, based on the available evidence and TASC scoring, MUC5AC and MUC16 should be considered biologically interesting candidates rather than leading translational targets.

#### 3.5.6. uPAR

The urokinase plasminogen activator receptor (uPAR) is a glycosylphosphatidylinositol (GPI)-anchored receptor that regulates extracellular matrix degradation, tumour angiogenesis, and metastasis [26,50]. uPAR is overexpressed in 67–80% of PDAC tissues, including neoplastic and stromal cells, with higher expression associated with poor survival outcomes [75]. While its expression in the tumour microenvironment enhances imaging potential, high staining in lymph node-negative tissues limits its specificity for detecting metastatic spread [11,24,28]. Nonetheless, uPAR remains a promising prognostic marker for disease staging and survival prediction.

#### 3.5.7. Mesothelin

Mesothelin (MSLN), a GPI-anchored adhesion protein in mesothelial cells, is a target for emerging PDAC-specific therapies, including antibody-based immunotherapy, CAR-T cell therapy, and vaccines [76,77,78]. Mesothelin is overexpressed in >90% of PDAC tissues, with negligible expression in normal pancreatic tissues [25]. It is involved in tumour cell migration, proliferation, and resistance to apoptosis, correlating with advanced disease and poor differentiation [71,77]. Low expression of MSLN observed in IHC and RT-PCR analysis of NPT and treatment-associated pancreatitis (TAP) provides evidence of MSLN’s potential to be used pre-operatively and for NAT-subjected tissues [25,33]. TASC scoring identifies MSLN as one of the most promising imaging biomarkers (score of 21), though further studies are needed to optimise its utility in early-stage disease detection and LNMs.

## 4. Discussion

This systematic review identified and evaluated tissue biomarkers in PDAC with potential relevance to tumour-targeted FGS. The findings suggest that several biomarkers have favourable biological and translational characteristics for intraoperative imaging, including membrane or extracellular localisation, relatively high tumour expression, and comparatively limited expression in normal tissues. Overall, the key finding of this review is not that a single optimal biomarker has already emerged, but that only a small proportion of PDAC biomarker research has progressed into clinically meaningful translational platforms capable of informing real-time surgical decision making. Therefore, biomarker expression should be interpreted in the context of surgical utility, including the ability to identify occult metastatic disease, define tumour boundaries, guide assessment of high-risk margins, and distinguish malignant infiltration from inflammatory or fibrotic tissue. Accordingly, the limited number of eligible studies is itself an important finding, demonstrating that relatively few candidate biomarkers have moved beyond expression-based discovery into platforms capable of addressing clinically relevant intraoperative problems.

Among the biomarkers identified, EGFR, MSLN, CEA, and integrin αvβ6 appear particularly attractive because of their tumour-associated expression patterns and biological accessibility. Antibody-based imaging agents have been developed against MSLN and integrin αvβ6 to enhance PDAC staging and detection [40,41,42], while fluorescent anti-EGFR and anti-CEA approaches have entered phase I/II clinical trials for FGS in pancreatic cancer [15,16]. In these studies, intraoperative fluorescence imaging improved visualisation of primary tumours compared with surrounding normal tissue and enabled detection of metastatic lymph nodes and small peritoneal deposits that were not readily apparent using conventional techniques [15,16]. This is clinically important because the value of FGS in PDAC is unlikely to lie simply in locating the primary tumour, which is usually apparent from preoperative imaging and operative anatomy, but rather in identifying otherwise occult disease and improving the accuracy of intraoperative staging and margin assessment. EGFR is additionally relevant because of its established role in tumour proliferation, migration and therapeutic targeting, including through clinically used anti-EGFR agents such as cetuximab, panitumumab, and related antibodies [79,80]. The current evidence remains insufficient to demonstrate that biomarker-targeted fluorescence can reliably change intraoperative decision-making, reduce positive resection rates, or improve recurrence outcomes. Future studies therefore need to move beyond proof-of-expression and should prospectively evaluate whether a fluorescence signal alters operative strategy, correlates with histopathological margins, identifies otherwise occult disease, and ultimately improves patient-centred oncological outcomes.

The clinical requirements for a FGS biomarker differ from those of a diagnostic, prognostic, or serum biomarker. For intraoperative use, a target must not only be overexpressed in tumour tissue, but must also provide reliable tumour-to-background contrast, spatially meaningful localisation, accessibility to a fluorescent probe, and sufficient contrast against adjacent normal pancreas, bile duct, duodenum, stomach, liver, lymph nodes, pancreatitis, and fibrosis [81]. This is particularly important in PDAC, where the tumour microenvironment is characterised by dense desmoplasia, heterogeneity, inflammation, and treatment-related stromal change [6].

The potential clinical value of biomarker-targeted FGS should also be considered in relation to existing intraoperative techniques. At present, pancreatic cancer surgery relies primarily on visual inspection, palpation, preoperative imaging, selective staging laparoscopy, intraoperative ultrasound, and frozen section analysis. These approaches remain essential, but each has important limitations. Visual inspection and palpation are subjective and may be less informative in minimally invasive surgery, after neoadjuvant therapy, or in the presence of pancreatitis and fibrosis. Frozen section analysis provides histological confirmation, but it is limited by sampling error, time delay, operator dependence, and the inability to assess the entire surgical bed [82]. A recent integrative review highlighted that there is currently no single gold standard intraoperative method for pancreatic cancer staging or margin assessment, and that frozen section and intraoperative ultrasound remain among the few established tools available to pancreatic surgeons for intraoperative assessment [7].

Artificial intelligence may also play an important role in the future development of tumour-targeted FGS. AI-based approaches could assist biomarker selection by integrating transcriptomic, proteomic, immunohistochemical and spatial datasets to prioritise targets with high tumour specificity and low background expression in normal or inflamed pancreatic tissue [83]. During surgery, AI could support fluorescence image interpretation through automated tumour segmentation, tumour-to-background quantification, autofluorescence correction, and real-time classification of suspicious tissue [84]. Ultimately, integration of fluorescence imaging with operative video, preoperative radiology, and predictive margin models may support intraoperative decision making. However, such systems require external validation and prospective clinical assessment before adoption into routine pancreatic cancer surgery. Recent reviews have specifically highlighted the potential synergy between fluorescence imaging and AI for precision cancer surgery and intraoperative decision support, although clinical validation remains limited [85]. For pancreatic cancer, AI-based histopathological and diagnostic approaches are also increasingly being explored for biomarker discovery and clinical decision support, reinforcing the relevance of incorporating computational methods into future FGS pipelines [86]. However, these systems must be externally validated, compatible with surgical workflow, and evaluated prospectively before they can be relied upon for intraoperative decision making.

This review highlights the numerous challenges and limitations inherent in the study of PDAC tissue biomarkers. One major challenge is the heterogeneity of biomarker expression across studies. Differences in tissue sampling methods (e.g., whole tissue sections vs. tissue microarrays) and variability in staining protocols hinder cross-study comparisons and limit the ability to perform meta-analyses. Lack of standardisation in the thresholds for biomarker overexpression further complicates biomarker validation and impacts reproducibility. Another challenge is the geographical bias in the literature, with many studies conducted in Eastern populations, making it difficult to generalise findings to Western PDAC patients. Biomarkers can vary between different ethnic groups, and studies conducted in specific populations may reflect regional or ethnic-specific variation. This is an important consideration for clinical applicability, as some biomarkers may exhibit differing sensitivities and specificities across diverse populations. Neoadjuvant therapy-related changes in biomarker expression, as seen with CEA and uPAR [24], may reduce diagnostic accuracy after treatment, presenting a barrier to their use in disease surveillance. Additionally, most studies are single-centre with limited multi-centre validation, restricting the generalisability of results. The incomplete availability of biomarker expression data in public repositories such as the Human Protein Atlas further impedes biomarker validation. Finally, methodological concerns arise from incomplete blinding and the absence of appropriate control tissues in some biomarker analyses. Addressing these challenges requires standardised tissue handling, multi-centre studies, and dual-marker approaches to improve the accuracy and reliability of PDAC biomarker research.

The findings of this review should be interpreted in the context of the variable methodological quality of the included biomarker studies. Although eight studies were judged to have low risk of bias, the remaining five studies were rated as having moderate risk of bias using the adapted REMARK framework. The main limitations related to incomplete reporting of patient selection, clinicopathological characteristics, antibody or reagent details, assay protocols, scoring thresholds, blinding of biomarker assessment, statistical analysis, and acknowledgement of potential sources of bias. These factors may affect the reproducibility of reported biomarker expression patterns and limit direct comparison between studies. This is particularly relevant for FGS, where candidate biomarkers require consistent tumour expression, low background expression in adjacent normal tissue, reproducible assay performance, and clear evidence of target accessibility. Therefore, although several promising PDAC tissue biomarkers were identified, further validation in well-characterised independent cohorts is required before clinical translation. Future studies should use standardised staining protocols, predefined scoring systems, blinded assessment by independent observers, complete reporting of antibody and assay details, and validation of tumour-to-background expression relevant to targeted fluorescence imaging.

## 5. Strengths and Limitations

This review has several strengths. It focused specifically on tissue-based and translational biomarkers relevant to FGS in PDAC, rather than broader diagnostic, serum, or genomic biomarkers without direct intraoperative applicability. The use of a structured TASC framework allowed biomarkers to be evaluated against translational criteria relevant to surgical imaging rather than expression level alone. The review also highlights an important gap between biomarker discovery and clinical implementation, emphasising the need for future studies to evaluate not only target expression but also imaging performance, tumour-to-background contrast, safety, timing, workflow compatibility, and impact on surgical decision making.

Several limitations, however, should be acknowledged. First, although MEDLINE/PubMed, Embase, and the Cochrane Library were searched, Scopus and Web of Science were not included. This may have limited retrieval of some multidisciplinary or citation-indexed records. However, the search strategy covered the major biomedical and clinical databases most relevant to the review question and was supplemented by clinical trial registry searches. Second, a formal inter-reviewer agreement statistic was not calculated for data extraction. To minimise inconsistency, data were extracted using a predefined form, cross-checked by multiple reviewers, and disagreements were resolved by consensus, with senior author adjudication where required. Third, although REMARK was used to reassess biomarker reporting quality, many included studies were not designed as formal prognostic or diagnostic accuracy studies. The REMARK framework was therefore applied as a structured tool for assessing reporting completeness and methodological transparency, rather than as a strict risk-of-bias instrument. Finally, the small number of included studies should be interpreted in the context of the narrow translational focus of this review. Although 6251 records were identified, most did not evaluate tissue-based PDAC targets suitable for active tumour targeting or FGS. Exclusion of cell line-only studies strengthened the clinical relevance of the review by prioritising biomarkers assessed in human PDAC tissue or translationally relevant platforms. However, this may have excluded early-stage mechanistic evidence, including target discovery studies, receptor binding experiments, internalisation assays, and preliminary fluorescent probe validation. Consequently, some emerging targets may not have been captured if they had not yet progressed to validation in primary PDAC tissue or clinically relevant models.

## 6. Conclusions

This systematic review demonstrates that several PDAC tissue biomarkers have potential relevance for tumour-targeted FGS, with EGFR, MSLN, CEA, and integrin αvβ6 showing particularly promising translational features, and MUC1 remaining the most relevant mucin-associated target within the TASC framework. However, the current evidence remains clinically immature. The next phase of research should prioritise head-to-head biomarker comparison, standardised tumour-to-background assessment, validation in post-neoadjuvant specimens, comparison with existing intraoperative techniques, and prospective evaluation of whether fluorescence-guided information changes surgical management or improves patient outcomes. Until such data are available, biomarker-targeted FGS should be regarded as a promising adjunct to, rather than a replacement for, established intraoperative assessment in pancreatic cancer surgery.

## Figures and Tables

**Table 1:** Inclusion and exclusion criteria for selecting studies on tissue biomarkers for PDAC. Inclusion criteria define the eligible population, sample type, biomarker characteristics, and outcome data.

Category	Inclusion Criteria	Exclusion Criteria
Population/disease	Studies assessing PDAC or pancreatic cancer with extractable PDAC-specific data.	Studies on metastatic malignancies to the pancreas originating from non-pancreatic primary sites, or mixed tumour cohorts from which PDAC-specific data could not be extracted.
Sample type	Studies assessing human PDAC tissue samples, including resection specimens, biopsy specimens, or tissue microarrays.	Studies evaluating only body fluids (e.g., serum, plasma, urine, pancreatic juice, or bile) without tissue-based assessment; studies based only on cancer cell lines or animal models without validation in primary human PDAC tissue.
Biomarker/target type	Studies reporting tissue-based biomarkers, molecular markers, receptors, antigens, or targets with potential relevance to tumour targeting, targeted imaging, fluorophore binding, or fluorescence-guided surgery.	Studies assessing biomarkers exclusively at the genetic or transcriptomic level (e.g., mutations, copy number alterations, or mRNA expression) without protein-level tissue expression data; studies assessing exclusively intracellular or nuclear markers inaccessible for extracellular molecular targeting.
Outcome/relevance	Studies providing extractable data on biomarker expression, localisation, tumour specificity, tumour-to-normal tissue contrast, or relevance to targeted imaging/FGS.	Studies reporting only clinical outcomes (e.g., survival, treatment response, or prognostic associations) without extractable tissue biomarker expression or target localisation data relevant to imaging.

Eligibility was further restricted to full-text, English-language primary research articles published between 1980 and January 2026. These are basic inclusion conditions rather than paired exclusion criteria; records not meeting them (e.g., non-primary publications, conference abstracts without full data, or duplicates) failed the inclusion criteria and were removed during screening, as reflected in Figure 1.

**Table 2:** Summary of studies included in the systematic analysis.

Biomarker(s)	Author (Year) [Reference]	Study Method	Stage	Cohort Size (n)	Tissue	PDAC (n)	Method of Analysis	Other Tissue Types Included	QA
Lea/c/x, sdi-Lea, sLea, sLex, sTn, MUC-1, MUC-5AC	Houvast et al. (2021) [23]	Retrospective	TNM	62	Human	48	TS/IHC	CP (n = 28) NPT (n = 31) HDT (n = 10)	Low Risk
Integrin αvβ6, CEACAM5, MSLN, PSMA, uPAR, FAP, ITGA5, EGFR	Vuijk et al. (2020) [24]	Retrospective	N/A	32	Human	32	TS/IHC	TAP (n = 15) NPT (n = 32)	Moderate Risk
MSLN	Le et al. (2020) [25]	Retrospective	TNM	24	Human	24	IHC	N/A	Low Risk
uPAR	de Geus et al. (2017) [26]	Retrospective	TNM	137	Human	122	TMA/IHC	N/A	Low Risk
Integrin αvβ6, CEACAM5, Cath E, EGFR, c-MET, Thy-1, uPAR	Tummers et al. (2017) [27]	Retrospective	TNM	1st cohort (n = 62), 2nd cohort (n = 55)	Human	1st cohort PDAC (n = 9) 2nd cohort PDAC (n = 12)	TS/IHC/ IF	1st cohort NPP (n = 8), CP (n = 7); 2nd cohort CP (n = 12), NPT (n = 12)	Low Risk
Integrin αvβ6, CEA, EGFR, uPAR	de Geus et al. (2016) [28]	Retrospective	TNM	165	Human	137	TMA/ IHC	PAAC (n = 28) NPT (n = 9)	Low Risk
EGFR	Guo et al. (2016) [29]	Retrospective	N/A	357	Human	357	TS/TMA/IHC	N/A	Low Risk
EGFR	Park et al. (2015) [30]	Retrospective	TNM	81	Human	81	TMA/IHC	NPT (n = 27), PanIN-1A (n = 16), PanIN-1B (n = 18), PanIN-2 (n = 11), PanIN-3 (n = 24)	Low Risk
EGFR, E-cadherin, β-catenin	Handra-Luca et al. (2014) [31]	Retrospective	TNM	99	Human	99	TMA/IHC	N/A	Moderate Risk
MUC-1, MUC-4	Remmers et al. (2013) [32]	Retrospective	N/A	44	Human	14	TMA/IHC	NPT (n = 8)	Low Risk
MSLN	Argani et al. (2001) [33]	Retrospective	TNM	60	Human	60	SAGE/IHC/ISH/RT-PCR	N/A	Moderate Risk
CEA	Allum et al. (1986) [34]	Retrospective	N/A	30	Human	30	IHC	CP (n = 10) NPT (n = 10)	Moderate Risk
CA19-9	Haglund et al. (1986) [35]	Retrospective	N/A	55	Human	55	TS/IHC	NPT (n = 29) CP (n = 23) PT (n = 12)	Moderate Risk

Abbreviations (in alphabetical order): CA19-9, carbohydrate antigen 19.9; CEA, carcinoembryonic antigen; c-MET, hepatocyte growth factor receptor; Cath E, cathepsin E; CP, chronic pancreatitis; EGFR, epidermal growth factor receptor; FAP, fibroblast activation molecule; HDT, healthy duodenal tissue; IF, immunofluorescence; IHC, immunohistochemistry; ISH, in situ hybridization; ITGA5, integrin alpha-5; MSLN, mesothelin; MUC, mucin; n, cohort or sample size; NPT, normal pancreatic tissue; N/A, not available; PanIN, pancreatic intraepithelial neoplasia; PDAC, pancreatic ductal adenocarcinoma; PAAC, peri-ampullary adenocarcinoma; PSMA, prostate-specific membrane antigen; PT, primary tumour; QA, Quality Assessment, RT-PCR, reverse transcription-polymerase chain reaction; SAGE, serial analysis of gene expression; TAP, tumour associated pancreatitis; Thy-1, thymus cell antigen-1; TMA, tissue microarray; TNM, tumour, node, and metastasis staging; TS, tissue specimen; uPAR, urokinase-type plasminogen activator receptor.

**Table 3:** Study characteristics of selected tissue biomarkers for PDAC.

Biomarker [Reference]	Main Outcomes	LN Detection	Location of Markers	Dual Biomarker Panel Analysis (Y/N)	Expression % in PDAC Tissue	Survival Outcome	NPT Expression
Lea/c/x, sdi-Lea, sLea, sLex, sTn, MUC-1, MUC-5AC [23]	All markers showed high overexpression on PDAC tissue. sLea and MUC-1 were simultaneously expressed in 94% of patients (dual tracers)	Lea/c/x → 90% sdi-Lea → 81% sLea → 81% sLex → 84% sTn → 81% MUC-1 → 97% MUC-5AC → 91%	Membrane-bound	Y	Lea/c/x → 83% sdi-Lea → 94% sLea → 98% sLex → 90% sTn → 88% MUC-1 → 96% MUC-5AC → 67%	NA	MUC-1 moderate to high expression on NPT. Others showed low NPT expression
Integrin αvβ6, CEACAM5, MSLN, PSMA, uPAR, FAP, ITGA5, EGFR [24]	Integrin αvβ6, CEACAM5, MSLN showed the highest expression in PDAC compared to TAP and NPT	Integrin αvβ6 → 100% CEACAM5 → 83% MSLN → 67%	Membrane-bound	N	integrin αvβ6 → moderate CEACAM5 → high MSLN → high PSMA → weak uPAR → weak FAP → weak ITGA5 → weak EGFR → weak	NA	Cohort of only NAT tissue analysis. Data reported as TNR with no pre-NAT biomarker expression analysis: Integrin αvβ6 → TNR = 4.1 CEACAM5 → TNR = 28.5 MSLN → TNR = 25.5
MSLN [25]	MSLN exhibited diffuse, high expression in PDAC with no expression in NPT	Not included	Membrane-bound	N	MSLN → 91.67%	NA	No expression in adjacent normal tissue
uPAR [26]	uPAR showed moderate to high expression in LN+, neoplastic, and stromal cells.	uPAR → 76%	Membrane-bound	N	uPAR → 66–82%	Predictive of OS/DFS	Low expression in NPT
Integrin αvβ6, CEACAM5, Cath E, EGFR, c-MET, Thy-1, uPAR [27]	Integrin αvβ6, CEACAM5, CathE, EGFR, and uPAR were significantly overexpressed in PDAC vs. CP Integrin αvβ6 specifically differentiates LN+ and LN- nodes. Low expression in CEACAM5 staining post-NAT	Integrin αvβ6 → 84%, CEACAM5 → 68%, EGFR → 93% Cath E → 54% uPAR → 69%	Membrane-bound	N	integrin αvβ6 → high CEACAM5 → high Cath E → high EGFR → high uPAR → high	NA	Integrin αvβ6 → high on NPT EGFR → moderate expression on HDT uPAR → high in normal LN
Integrin αvβ6, CEA, EGFR, uPAR [28]	All 4 markers showed high expression in PDAC tissue	Integrin αvβ6 → 96% CEA → 96% c-MET → 96% EGFR → 95% EpCAM → 92% HER2 → 92% VEGFR2 → 94% uPAR → 92%	Membrane-bound	Y	Integrin αvβ6 → 86% CEA → 71% EGFR → 96% uPAR → 67%	NA	All 4 markers showed low expression in NPT
EGFR [29]	EGFR expression strongly correlated with adjuvant treatment outcome	Not included	Membrane-bound	N	EGFR → 54.6%	No correlation with OS	NA
EGFR [30]	High expression of EGFR in PDAC and high-grade PanIN3	Not included	Membrane-bound	N	EGFR → 64.2–81%	No correlation with OS	No expression in NPT, PanIN-1A, and PanIN-1B
EGFR, E-cadherin, β-catenin [31]	High expression of all biomarkers in PDAC	High expression of all biomarkers in +LN	Membrane-bound	Y	EGFR → 62% E-cadherin → 98% β-catenin → 94%	No correlation with OS	NA
MUC-1, MUC-4 [32]	High expression of MUC-1/4 in PDAC	Not included	Membrane-bound	N	MUC-1 → 66%	NA	High expression of MUC-1/4 in NPT
MSLN [33]	Intense granular cytoplasmic labelling with the mesothelin antisense riboprobe	Not included	Membrane-bound	N	MSLN → 90%	NA	There was no MSLN labelling in any PanIN lesions. Weakly labelled in 8/60 (13%) cases of CP
CEA [34]	High expression of CEA in PDAC and luminal surfaces of glandular neoplastic tissue	Not included	Membrane-bound	N	CEA → 77%	NA	High CEA expression in CP. No expression in NPT
CA19-9 [35]	High expression in PDAC and CP	Not included	Membrane-bound	N	CA19-9 → 85% CA19- → 96% (CP)	NA	High CA19-9 expression in CP → 96% High CA19-9 expression in NPT adjacent to CP or PDAC

Abbreviations (in alphabetical order): CA19-9, carbohydrate antigen 19.9; CEA, carcinoembryonic antigen; c-MET, hepatocyte growth factor receptor; CP, chronic pancreatitis; DFS, disease free survival; EGFR, epidermal growth factor receptor; EpCAM, epithelial cell adhesion molecule; FAP, fibroblast activation molecule; HDT, healthy duodenal tissue; HER2, human epidermal growth factor receptor 2; ITGA5, integrin alpha-5; LN, lymph node; LN+, lymph node positive; LN-, lymph node negative; MSLN, mesothelin; MUC, mucin; N, no; NAT, neoadjuvant therapy; NPT, normal pancreatic tissue; NA, not available; OS, overall survival; PanIN, pancreatic intraepithelial neoplasia; PDAC, pancreatic ductal adenocarcinoma; PSMA, prostate-specific membrane antigen; TAP, tumour associated pancreatitis; Thy-1, thymus cell antigen-1; TNR, tumour-to-normal tissue ratio; uPAR, urokinase-type plasminogen activator receptor; VEGFR2, vascular endothelial growth factor receptor 2; Y, yes. The tumour-to-normal tissue ratio (TNR) is a quantitative parameter in medical imaging that assesses relative signal intensity between tumour tissue and adjacent normal tissue. Higher TNR values indicate enhanced contrast and improved tumour delineation.

**Table 4:** Summary of reported clinical trials for targeted FGS in PDAC.

Study Biomarker	Therapeutic Antibody	Study Type	Emission Wavelength of the Fluorophore	Number of Patients	TNR (Mean)	Metastatic Lesions (Mean)	Conclusions
EGFR [15]	Panitumumab (IRDye800CW)	Phase I (dose-escalation)	789 nm	11	3.0, 4.0, and 3.7 for the 25, 50, and 75 mg groups, respectively	AUC 0.87 (95% CI: 0.83–0.91) Sensitivity of 67.2% (95% CI: 57.8–80.6%) Specificity 92.1% (95% CI: 89.3–94.4%)	Panitumumab-IRDye800CW was safe and feasible to use for FGS.
CEA [16]	SGM-101	Phase I	704 nm	12	1.6 ± 0.37	1.7 ± 0.42	The use of a fluorescently- labelled anti-CEA antibody was safe and feasible. One false-positive lesion (CEA-expressing IPMN). Two false-negatives: overlying blood or tissue that blocked the fluorescent signal.
EGFR [36]	Cetuximab-IRDye800	Phase I	800 nm	10	2.3 ± 0.72	6.3 ± 0.82	Cetuximab-IRDye800 allowed significantly higher mean fluorescence intensity in the tumour compared with normal pancreatic tissue and pancreatitis (*p* < 0.001).
VEGF [37]	Bevacizumab-IRDye800CW	Phase I (dose-escalation)	700 nm	10	1.4 (n = 2), 1.7 (n = 1), and 2.1 (n = 1) in the 4.5, 10, and 25 mg dose groups, respectively	NA	Bevacizumab-IRDye800CW was feasible and safe; however, tumour-to-background fluorescence contrast was limited

Abbreviations (in alphabetical order): AUC, area under the curve; CEA, carcinoembryonic antigen; CI, confidence interval; EGFR, epidermal growth factor receptor; FGS, fluorescence-guided surgery; IPMN, intraductal papillary mucinous neoplasm; NA, not available/not applicable; n, number of patients or samples; *p*, probability value; PDAC, pancreatic ductal adenocarcinoma; TNR, tumour-to-normal tissue ratio; VEGF, vascular endothelial growth factor.

**Table 5:** Theranosis (TASC-T) scoring criteria for PDAC tissue biomarkers [39].

No.	Parameter	Score
1	Extracellular or membrane localisation of biomarker	5
2	20% positivity in tumour tissue	4
3	Low expression in normal pancreatic tissue	3
4	Percentage positivity or upregulation in tumour tissue	
	>90%	6
	70–90%	5
	50–69%	3
	<49%	0
5	Previous application to imaging	
	preclinical	1
	clinical	2
6	Ligand in human trials	1
	Total	21

**Table 6:** TASC-T scoring of best-performing biomarkers in this systematic review.

Tumour Biomarker	Extracellular Localisation	Membrane Localisation	>20% Positivity in Tumour Tissue	Low Expression in Normal Pancreatic Tissue	Highest % Positivity/ Upregulation Reported in PDAC	Ligand in Human Trials	Previous Use in Pre-Clinical Imaging	Previous Use in Clinical Imaging [Reference]	TASC-T (Max 22)
EGFR	Yes	Yes	Yes	Yes	60–96	Yes	Yes	Yes [10]	21
MSLN	Yes	Yes	Yes	Yes	>90	Yes	Yes	Yes [40]	21
Integrin αvβ6	Yes	Yes	Yes	Yes	86	Yes	Yes	Yes [41,42]	20
CEA	Yes	Yes	Yes	Yes	77	Yes	Yes	Yes [11]	20
uPAR	Yes	Yes	Yes	Yes	82	No	Yes	No	18
MUC-1	Yes	Yes	Yes	No	96	Yes	Yes	No	17
CA19.9	Yes	Yes	Yes	No	85	Yes	Yes	Yes [43]	17

## Data Availability

Data extracted for this systematic review will be provided to interested parties upon reasonable request to the corresponding author.

## Supplementary Materials

The following supporting information can be downloaded at: https://www.mdpi.com/article/10.3390/cimb48070717/s1.
