# Supplementary material for: Tissue-Based Biomarkers for Fluorescence-Guided Surgery of Pancreatic Ductal Adenocarcinoma: A Systematic Review
Source: Curr Issues Mol Biol. 2026 Jul 14;48(7):717. doi: 10.3390/cimb48070717 (PMC13409608; doi:10.3390/cimb48070717)
Supplement: Supplementary file 1 [file cimb-48-00717-s001.zip › cimb-4315792-supplementary-proof.pdf]

Supplementary Table S1:

The methodological quality of the 13 included studies was assessed using an adapted REMARK framework, modified to reflect the biomarker expression, prognostic, and target-validation designs of the included papers. Thirteen reporting domains were evaluated for each study: patient cohort description, eligibility criteria, clinicopathological data, tissue specimen source and handling, biomarker assay method, antibody and reagent details, scoring and cut-off method for biomarker positivity, blinding or independent assessment, outcome or target-validation endpoint, statistical analysis, confounder adjustment (where applicable), completeness of results, and acknowledgement of limitations or sources of bias. Ratings: Yes = clearly reported / low concern (score 2); Partial = incompletely reported / some concern (score 1); No = not reported / high concern (score 0); N/A = not applicable (excluded from denominator). A total score and percentage of fulfilled items were calculated for each study based on applicable domains only. An overall risk-of-bias judgement was assigned using equal-interval thresholds: Low risk (>67% of applicable domains fulfilled), Moderate risk (34–67%), and High risk (<34%). Conservative ratings were applied: where critical methodological details were absent from the main text and deferred to supplementary files, the domain was rated Partial rather than Yes.

Supplementary Table S1

REMARK-based quality assessment of included PDAC tissue biomarker studies.

Study	Cohort described	Eligibility criteria	Clinico-pathological data	Tissue handling	Assay method	Reagent details	Scoring/ cut-off	Blinding/ independent review	Endpoint defined	Statistical analysis	Confounders considered	Results complete	Bias/ limitations	Score (max)	% Fulfilled	Overall judgement
**Houvast et al., 2021**	Yes	Yes	Yes	Yes	Partial	Partial	Yes	No	Yes	Yes	N/A	Yes	Yes	**20/24**	**83%**	**Low risk**
**Vuijk et al., 2020**	Partial	Partial	Partial	Partial	Partial	Partial	Partial	Partial	Yes	Yes	N/A	Yes	Yes	**16/24**	**67%**	**Moderate risk**
**Le et al., 2020**	Yes	Yes	Partial	Partial	Partial	Yes	Yes	Yes	Yes	Yes	N/A	Yes	No	**19/24**	**79%**	**Low risk**
**Tummers et al., 2017**	Yes	Yes	Partial	Yes	Yes	Partial	Yes	Yes	Yes	Yes	N/A	Yes	Yes	**22/24**	**92%**	**Low risk**
**de Geus et al., 2017**	Yes	Yes	Yes	Yes	Yes	Yes	Yes	Yes	Yes	Yes	Yes	Yes	Yes	**26/26**	**100%**	**Low risk**
**Guo et al., 2016**	Yes	Yes	Yes	Yes	Partial	Partial	Yes	Yes	Yes	Yes	Yes	Yes	No	**22/26**	**85%**	**Low risk**
**de Geus et al., 2016**	Yes	Yes	Yes	Yes	Yes	Partial	Yes	Yes	Yes	Yes	N/A	Yes	Yes	**23/24**	**96%**	**Low risk**
**Park et al., 2015**	Yes	Partial	Partial	Yes	Partial	Partial	Yes	No	Yes	Yes	Yes	Yes	No	**18/26**	**69%**	**Low risk**
**Remmers et al., 2013**	Partial	Partial	Partial	Yes	Yes	Partial	Yes	Partial	Yes	Yes	N/A	Yes	No	**17/24**	**71%**	**Low risk**
**Handra-Luca et al., 2014**	Partial	Partial	Partial	Partial	Partial	Partial	Partial	No	Yes	Yes	Yes	Yes	No	**15/26**	**58%**	**Moderate risk**
**Argani et al., 2001**	Partial	Partial	Partial	Partial	Yes	Yes	Yes	Partial	Yes	No	N/A	Yes	No	**15/24**	**62%**	**Moderate risk**
**Allum et al., 1986**	Partial	Partial	Partial	Partial	Yes	Yes	Partial	Yes	Yes	No	N/A	Yes	No	**15/24**	**62%**	**Moderate risk**
**Haglund et al., 1986**	Partial	Partial	Partial	Partial	Yes	Partial	Partial	No	Yes	No	N/A	Yes	No	**12/24**	**50%**	**Moderate risk**

Table footnote

Assessment was based on relevant REMARK domains adapted for tumour biomarker expression and prognostic/target-validation studies. Studies are ordered from newest to oldest. Ratings: Yes = clearly reported / low concern (score 2); Partial = incompletely reported / some concern (score 1); No = not reported / high concern (score 0); N/A = not applicable (excluded from denominator). The percentage of fulfilled items was calculated as (total score / maximum applicable score) × 100. Overall risk-of-bias judgement was assigned using equal-interval thresholds: Low risk >67%; Moderate risk 34–67%; High risk <34%. Conservative ratings were applied throughout: where critical methodological details were absent from the main text and deferred to supplementary files not reproduced in the article, the domain was rated Partial rather than Yes.

Domain key (column abbreviations)

**Cohort described**	Patient population clearly described (sample size, tumour type, institution, time period).
**Eligibility criteria**	Inclusion and exclusion criteria explicitly stated in the main text.
**Clinicopathological data**	Patient demographics and tumour characteristics (age, sex, TNM stage, grade, LN status, margins) reported.
**Tissue handling**	Tissue source, fixation method, and preparation protocol described in the main text.
**Assay method**	IHC or other assay protocol described in sufficient detail (antigen retrieval, secondary antibody, detection system, counterstain).
**Reagent details**	Primary antibody clone, catalogue number, supplier, and dilution explicitly stated in the main text.
**Scoring/cut-off**	Method and threshold for defining biomarker positivity clearly described.
**Blinding/independent review**	Histological scoring performed blinded to clinical outcomes and/or by ≥2 independent observers.
**Endpoint defined**	Primary study endpoint clearly stated (expression rate, OS, DFS, TNR, LN detection sensitivity, etc.).
**Statistical analysis**	Statistical tests named, significance threshold stated, and appropriate for data type.
**Confounders considered**	Multivariable analysis performed to adjust for confounders (prognostic studies); N/A for expression-only studies.
**Results complete**	All pre-specified outcomes reported, including negative or null findings.
**Bias/limitations**	Study limitations and sources of bias explicitly acknowledged.
**Score (max)**	Total score / maximum applicable score (Yes=2, Partial=1, No=0; N/A excluded from denominator).
**% Fulfilled**	Percentage of fulfilled items = (total score / maximum applicable score) × 100.
